# Enzyme Selection and Hydrolysis under Optimal Conditions Improved Phenolic Acid Solubility, and Antioxidant and Anti-Inflammatory Activities of Wheat Bran

**DOI:** 10.3390/antiox9100984

**Published:** 2020-10-13

**Authors:** Sara Bautista-Expósito, Irene Tomé-Sánchez, Ana Belén Martín-Diana, Juana Frias, Elena Peñas, Daniel Rico, María Jesús García Casas, Cristina Martínez-Villaluenga

**Affiliations:** 1 Institute of Food Science, Technology and Nutrition (ICTAN-CSIC), José Antonio Novais, 10, 28040 Madrid, Spain; sara.bautista@ictan.csic.es (S.B.-E.); i.tome@ictan.csic.es (I.T.-S.); frias@ictan.csic.es (J.F.); elenape@ictan.csic.es (E.P.); 2 Agricultural Technological Institute of Castile and Leon (ITACyL), Government of Castile and Leon. Ctra. de Burgos Km. 119, Finca Zamadueñas, 47071 Valladolid, Spain; mardiaan@itacyl.es (A.B.M.-D.); ricbarda@itacyl.es (D.R.); garcasma@itacyl.es (M.J.G.C.)

**Keywords:** wheat bran, enzymatic hydrolysis, Ultraflo XL, ferulic acid, antioxidant activity, anti-inflammatory activity

## Abstract

Valorization of wheat bran (WB) into new high-value products is of great interest within the framework of sustainability and circular economy. In the present study, we utilized a multi-step approach to extract nutraceutical compounds (phenolic acids) from WB and improved its antioxidant and anti-inflammatory properties through using sequential hydrothermal and enzymatic hydrolysis. Thirteen commercial glycosidases differing in their specific activity were screened and compared for hydrolytic efficiency to release monosaccharides, ferulic acid, and diferulic acid. Ultraflo XL was selected as the desired enzyme treatment on the basis of its higher WB solubilization, as well as its monosaccharide and phenolic acids yields. The relationships between better hydrolytic performance of Ultraflo XL and its particular activity profile were established. To determine the optimum conditions for Ultraflo XL treatment, we tested different factors (solvent pH, incubation temperature, and time) under 15 experiments. A multicomponent analysis (MCA), including central composite design, model fitness, regression coefficients, analysis of variance, 3D response curves, and desirability, was used for processing optimization. A beneficial effect of autoclave treatment on the release of phenolic compounds was also evidenced. The results of MCA showed involvement of linear, quadratic, and interactive effects of processing factors, although solvent pH was the main determinant factor, affecting enzymatic extraction of phenolics and bioactivity of hydrolysates. As compared to control WB, under optimized conditions (47 °C, pH = 4.4, and 20.8 h), WB hydrolysates showed 4.2, 1.5, 2, and 3 times higher content of ferulic acid (FA) and capacity to scavenge oxygen radicals, chelate transition metals, and inhibit monocyte chemoattractant protein-1 secretion in macrophages, respectively. These approaches could be applied for the sustainable utilization of WB, harnessing its nutraceutical potential.

## 1. Introduction

The agrifood industry has been undergoing a constant transformation that focuses on the recovery of by-products for the production of sustainable ingredients for food and beverages, feed, and the pharmaceutical industry. During the wheat grain milling process, the aleurone layer from the starchy endosperm remains mainly attached to the peripheral layers and is recovered in a technological fraction known as bran that constitutes between 10 and 15% of the kernel weight [1,2]. Wheat bran (WB) is considered a by-product of the cereal processing industry usually used as livestock feed [3], although there is an increasing trend in its use as a fiber-rich ingredient in food products [4]. WB is a dietary source of fiber (36.5–52.4% of dry weight) mainly composed by arabinoxylans (AX, 70%), cellulose (24%), and β-glucan (6%) [5]. Minor amounts of glucomannans, arabinogalactans, and xyloglucans have also been reported [6].

WB is also an important source of phytochemicals that may offer protection against chronic diseases [7]. Attending to their abundance and health benefits, phenolic compounds (1942–5400 mg/kg) are the most important phytochemicals in WB. They are mainly concentrated in the aleurone layer and outermost pericarp, as well as the testa tissues of the bran [8]. Ferulic acid (FA), the most abundant phenolic compound in WB, has shown antioxidant, anti-inflammatory, antihypertensive, and anti-arteriosclerotic effects in vitro and in vivo [9]. The accumulated scientific evidence of health benefits exerted by FA have promoted the interest of researchers to explore the potential use of WB as a resource in the production of functional ingredients [10].

In WB, around 92% of FA is linked to the arabinofuranosyl residues of AX and other cell wall structures while the remaining 8% can be found as a soluble-free compound or conjugated moiety esterified to sugars [11]. FA also cross-links the WB polysaccharides by different dimers, namely, 8-5′-, 5-5′-, 8-*O*-4′-, and 5-8′-diferulic acids (DFA), produced via the oxidative polymerization processes [12]. The fact that FA and DFA isomers are mainly bound to polysaccharides explain their low bioavailability in WB (<3%) compared to other plant matrices [13]. Different WB processing methods aiming to increase FA and DFA bioavailability have been explored including microbial fermentation, alkaline/acid, and enzymatic hydrolysis [14]. In the context of sustainable processing, enzymatic hydrolysis is particularly attractive because of its environmentally friendliness, greater energy efficiency, and higher selectivity when compared with other methods [15]. Additionally, enzymatic treatments do not extensively modify the initial organoleptic properties of WB.

Solubilization yield of FA and derivatives from WB matrix and the biological activity of the WB hydrolysate depend on the type of enzyme(s) and reaction conditions selected during enzymatic treatments (enzyme to substrate ratio, pH, temperature, time, etc.) [15]. Therefore, optimization of reaction conditions is also crucial to increase the biological efficiency of WB hydrolysates. The aim of this study was to solubilize the maximum amount of bound FA and DFAs from WB to obtain a functional ingredient with the highest bio-efficacy (maximum bioactivity) in terms of antioxidant and anti-inflammatory properties. To accomplish this objective, we screened 13 food-grade commercial enzymes for activity profile and ability to hydrolyze WB matrix on the basis of the release of monosaccharides and bound FA and DFAs. The effect of application of hydrothermal pretreatment on yields of soluble phenolic compounds was also evaluated. Finally, processing parameters such as pH, temperature, and time in the production of WB hydrolysates were further optimized through central composite design (CCD) of response surface methodology (RSM) for maximum solubilization of phenolic acids, as well as antioxidant and anti-inflammatory activities. A multi-response optimization method (desirability) was applied to identify the optimal processing parameters to enhance the nutraceutical value of WB hydrolysates.

## 2. Materials and Methods 

### 2.1. Materials

WB was kindly supplied by Emilio Esteban, S.A. (EMESA, Valladolid, Spain) and stored in plastic bags under vacuum until use. WB showed the following composition: 53.9 ± 3.3% of dietary fiber, 4.0 ± 0.5% of fat, 12.6 ± 0.2% of moisture, 13.1 ± 0.2% of protein, and 7.2 ± 0.0% of ash. Total dietary fiber was determined using the rapid integrated total dietary fiber assay procedure (Megazyme, Wicklow, Ireland). Moisture content was analyzed gravimetrically using drying treatment of the samples at 100 °C for 24 h. Fat content was measured using petroleum ether 40–60 °C for over 4 h of extraction and was gravimetrically determined. Nitrogen content was analyzed by the Dumas method, using a nitrogen analyzer (LECO Corp., St. Joseph, MI, USA). Ash content was determined by gravimetry following the Association of Official Agricultural Chemists (AOAC) method 923.03. The commercial food grade enzymes Depol 333MDP, Depol 40L, Depol 667P, Depol 670L, Depol 686L, Depol 740L, Depol 761P, Depol 793L, and Pectinase 62L were obtained from Biocatalyst Ltd. (Cardiff, Wales, United Kingodm). Shearzyme, Ultraflo XL, Viscoferm, and Viscozyme were acquired from Novozymes (Bagsværd, Copenhagen, Denmark). All chemicals of analytical grade were purchased from Sigma-Aldrich (Madrid, Spain), unless otherwise stated.

### 2.2. Enzymatic Activity Assays

Cellulase (CELase, EC 3.2.1.4) and xylanase (XYLase, EC 3.2.1.8) activity in commercial glycosidase cocktails were determined using K-CellG3 and XylX6 methods from Megazyme (Bray Co., Wicklow, Ireland). Results were expressed as units (U) per gram of enzymatic cocktail. One unit of CELase activity is defined as the amount of enzyme required to release 1 µmole of 2-chloro-4-nitrophenol from CellG3 in 1 minute at pH = 6.0 and 40 °C. One unit (U) of XYLase activity is defined as the amount of enzyme required to release 1 µmole of *p*-nitrophenol from the XylX6 in 1 minute at pH = 6 and 40 °C. α-l-Arabinofuranosidases (AFase, EC 3.2.1.55) and feruloyl esterase (FEase, EC 3.1.1.73) activities were determined following the procedure reported by Dupoiron et al. [16]. Briefly, 100 μL of diluted solutions of commercial enzymes in 0.1 M sodium phosphate buffer (pH 6.0) were incubated with 100 μL of 1 mM nitrophenyl-α-L-arabinofuranoside at 40 °C for 10 min and shaking speed of 1000 rpm in a Thermomixer (Eppendorf Iberica, Madrid, Spain). Absorbance was measured at 400 nm in a microplate reader (Biotek Instruments, Winooski, VT, USA) after addition of 1 mL of 2% Tris buffer (pH = 10). An external calibration curve of *p*-nitrophenol at a concentration range from 0.01 to 0.5 μmol/mL was prepared to calculate the amount of ferulate release per minute. Results were expressed as units per gram of enzymatic cocktail. One unit (U) of AFase activity is defined as the amount of enzyme required to release 1 µmole of *p*-nitrophenol per minute from *p*-nitrophenyl-α-l-arabinofuranoside (5 mM) in sodium acetate buffer (100 mM) at pH = 6.0 and 40 °C. FEase activity was carried out by dissolving enzymes and substrate in 100 mM 3-(N-morpholino)propanesulfonic acid (MOPS) buffer (pH = 6.0) containing 2.5% Triton X-100. A volume of 100 μL of enzyme solutions were incubated with 100 μL of 0.5 mM *p*-nitrophenol *trans*-ferulate (Carbosynth, Berkshire, United Kingdom) at 40 °C for 10 min. Absorbance was measured every minute at 320 nm in a microplate reader (Biotek Instruments, Winooski, VT, USA). An external calibration curve of FA of a concentration range from 0.02 to 0.3 μmol/mL was prepared to calculate the amount of ferulate released per minute. Results were expressed as milliunits per gram of enzymatic cocktail. One unit (U) of FEase activity is defined as the amount of enzyme required to release 1 µmole of ferulic acid from *p*-nitrophenyl *trans*-ferulate in 1 minute at pH = 6 and 40 °C.

### 2.3. Enzymatic Hydrolysis of WB

For enzyme selection experiments, we suspended WB (<1000 µm particle size) in 100 mM sodium phosphate buffer (pH = 6; 1:20 solid to solvent ratio). Enzymes were added into WB suspensions at 1% (enzyme to WB dry weight ratio, *w*:*w*). WB hydrolysis was performed under 40 °C conditions at 1000 rpm for 20 h using a hot-plate magnetic-stirrer device controlled by VTF EVO digital thermoregulators (VELP Scientifica, Madrid, Spain). WB suspensions without enzyme addition served as the control.

The hydrolysis conditions of the selected enzyme were further optimized under the following experimental design. WB suspensions (1:20 solid to liquid ratio) were firstly autoclaved at 121 °C, 1 atm for 30 min. Experimental design was set using Statistica 7.0 software (Statsoft, Tulsa, OK, USA) in which a CCD of 3 factors, 1 block, 16 runs, and 3 center points (for rotability and orthogonality) was chosen. Temperature, pH, and time were selected as independent factors in a range of 46.6 to 63.4 °C, 3.3–6.7, and 0.5–27.5 h, respectively. Experiments were performed in a randomized order to assure the independence of the results. Two buffer solutions were used for WB hydrolysis: 100 mM phosphate buffer for pH values from 6 to 6.7 and 100 mM sodium acetate buffer for pH values from 3.3 to 5.0. In all experimental runs, we added Ultraflo XL at 1% (enzyme to WB ratio, *w*/*w*). Reaction mixtures were stirred at 1,000 rpm and warmed using a hot-plate magnetic-stirrer device controlled by digital thermoregulators (VELP Scientifica, Madrid, Spain) during incubation time.

At the end of incubation period, we inactivated enzymes by submerging WB hydrolysates in a water bath at 95 °C for 10 min. Solid residues were removed by means of a nylon mesh filter (200 μm, Alcavida, Barcelona, Spain). Finally, filtrates were freeze-dried, and their dry weights were monitored and stored under vacuum at −20 °C until further analysis. The WB solubilization yield (Ys) was calculated as shown in Equation 1:

(1) $$Y_{0}=M_{p}/M_{0}\cdot 100$$

where $M_{p}$ is the dry weight (g) of the soluble fraction and $M_{0}$ is the weight of WB (g). Experimental assays were performed in triplicate.

### 2.4. Identification and Quantification of Monosaccharides

Monosaccharides were determined by high performance anion exchange chromatography in water-soluble extracts of WB and its hydrolysates. One gram of sample was extracted with 20 mL of distilled water at a temperature of 40 °C at 1000 rpm for 30 min in a thermomixer (Eppendorf Billerica, Madrid, Spain). Subsequently, samples were centrifuged at 10,000× *g* at 4 °C for 10 min using a 5424 R centrifuge (Eppendorf Billerica, Madrid, Spain). The supernatant was collected, and the extraction process was repeated once again. An ion chromatographic system coupled to a 800 Dosino dispenser, Bioscan module, and a pulse amperometric detector was used (Metrohm, Heriau, Switzerland). Data acquisition and processing were achieved by Metrodata IC Net 2.3. software. Monosaccharides were separated in a Hamilton RCX-30 column (4.1 × 250 mm, 7 μm) using 120 mM NaOH as eluent at a flow rate of 1 mL/min. The column temperature was maintained at 30 °C and the injection volume was 20 µL. Identification of monosaccharides was achieved by chromatographic comparison of retention time and sample spike with authentic standards. Quantification was done using external calibration curves of a multi-standard made up of d-(+)-glucose, d-(+)-galactose, d-(+)-xylose, and D-(−)-arabinose. Calibration curves showed good linearity among different concentrations of monosaccharides. The calibration plots revealed good correlation between peak areas and analyte concentrations, and the regression coefficient was higher than 0.99. Results were expressed as milligram of monosaccharide per gram of bran in dry basis.

### 2.5. Extraction and Quantification of Total Soluble Phenolic Compounds (TSPC)

The extraction of TSPC was conducted according to the method described by Dinelli et al. [17] with some modifications. One gram of sample was extracted with 20 mL of ethanol/water (80:20, *v*:*v*) at 4 °C for 10 min. Subsequently, samples were centrifuged at 2500× *g* at 4 °C for 10 min using a Sorval RC 5B centrifuge (ThermoFisher, Madrid, Spain). The supernatant was collected, and the extraction process was repeated once again. The extracts were pooled and evaporated in a rotavapor (Büchi Labortechinik, Flawil, Switzerland) at 175 psi and 40 °C. Dry extracts were dissolved in 2 mL of methanol/water (80:20, *v*:*v*). Finally, samples were stored at −20 °C until being used. TSPC in hydroalcoholic extracts were quantified by the Folin–Ciocalteu method [18]. Results were expressed as milligram of FA equivalents (FAE) per gram of bran or 100 g of hydrolysate (soluble fraction) in dry basis. 

### 2.6. Identification and Quantification of FA and Derivatives

In the screening experiments, we carried out HPLC analyses using an Agilent 1200 series high resolution liquid chromatograph equipped with a diode array detector model G1315B (Agilent Technologies, Santa Clara, CA, USA). The phenolic fractions were separated in a Novapack C18 analytical column (3.9 mm × 150 mm, 4 µm). The gradient eluent was used at flow rate of 0.8 mL/min. The column temperature was maintained at 25 °C and the injection volume was 10 µL. The mobile phases were 1% formic acid in water (A) and acetonitrile (B). The gradient elution was performed as follows: from 5% B to 60% B in 37 min, from 60% B to 98% B in 3 min, and from 98% B to 5% B in 5 min. In addition, the HPLC system was coupled to an accurate mass quadrupole TOF spectrometer using an electrospray interface with JetStream technology (model G6530A from Agilent Technologies, Santa Clara, CA, USA). Analysis parameters were set using a negative-ion mode with spectra acquired over a mass range from *m/z* 100 to 1500. The optimum values of the electrospray ionization mass spectrometry (ESI-MS) parameters were capillary voltage, +4.5 kV; drying gas temperature, 190 °C; drying gas flow, 9.0 L/min; and nebulizing gas pressure, 2 bar. The accurate mass data on the molecular ions were processed through Masshunter Data Acquisition B.05.01 software (Agilent Technologies, Santa Clara, CA, USA). Quantification of FA and derivatives was made according to the linear calibration curves of FA in the concentration range from 4.5 to 450 μg/mL of FA. Calibration curves showed good linearity and the regression coefficient was higher than 0.99. The results were expressed in micrograms FA equivalents per gram WB in dry weight basis (d.w.).

In the optimization experiments, we determined FA by reverse phase HPLC using an Alliance Separation Module 2695 (Waters, Milford, CT, USA) consisting of a vacuum degasser, autosampler, and a binary pump connected to a photodiode array detector (model 2996 from Waters, Milford, CT, USA). The phenolic compounds were separated in the same column and conditions specified above. Data acquisition and integration was performed using Empower II software (Waters, Milford, CT, USA). FA was identified by retention time, maximum absorbance, and spiking with a standard solution. External calibration curves of a standard solution of FA were built in a concentration range from 4.5 to 450 μg/mL. The results were expressed in milligram FA per 100 g of hydrolysate (soluble fraction) in dry basis.

### 2.7. Determination of Antioxidant Activity

The antioxidant activity was determined following 4 previously described in vitro methodologies: oxygen radical absorbance capacity (ORAC) [19], 2,2′-azino-bis-3-ethylbenzothiazoline-6-sulfonic acid (ABTS) radical cation [20], ferric reducing antioxidant power (FRAP) [21], and 2,2-diphenyl-1-picryl-hydrazyl-hydrate (DPPH) radical scavenging activity [22]. ORAC assay was performed at 37 °C in 75 mM phosphate buffer at pH = 7.4. The reaction mixture contained 180 μL of 70 nM fluorescein, 90 μL of 12 mM 2,2′-azobis(2-amidinopropane) dihydrochloride (AAPH), and 30 μL of diluted sample or the standard 6-hydroxy-2,5,7,8-tetramethylchroman-2-carboxylic acid (Trolox) at concentrations ranging from 1 to 160 μM. Reaction mixtures were placed in a black 96-well plate and fluorescence was read in a Synergy HT microplate reader (BioTek Instruments, Winooski, VT, USA) every minute at excitation and emission wavelengths of 485 and 520 nm, respectively. The equipment was controlled by Gen5 software, version 1.1 (BioTek Instruments, Winooski, VT, USA). Results were expressed as micromole Trolox equivalents (TE) per 100 g of hydrolysate soluble fraction in dry basis. ABTS assay was carried out mixing 200 µL of ABTS●+ working solution and 20 µL of diluted samples or Trolox (concentration range from 7.5 to 240 μM) in 96-well plates. The decay in absorbance at 734 nm was monitored over 30 min in a microplate reader (Spectrostar Omega, BMG, Ortenberg, Germany). Values were expressed as micromole TE/100 g of hydrolysate in dry weight basis. DPPH assay was performed by mixing 125 µL of 100 µM DPPH radical methanolic solution with 25 µL of sample extracts and 100 µL of bidistilled water in 96-well plates. Absorbance was measured at 515 nm every min for 30 min using a microplate reader (Spectrostar Omega, BMG, Ortenberg, Germany). Values were calculated from a standard calibration curve of Trolox (7.5–240 μM) and expressed as milligrams TE/100 g of hydrolysate soluble fraction in dry basis. FRAP assay was initiated by mixing 38 mM sodium acetate anhydrous at pH = 3.6, 20 mM Fe(III) Cl_3_, and 10 mM 2,4,6-tri(2-pyridyl)-*S*-triazine at a ratio of 10:1:1 (*v*:*v*:*v*). Reaction mixtures were kept at 37 °C for 40 min in heating blocks and covered with tin foil. The absorbance of the supernatant was monitored at 593 nm using a microplate reader (Spectrostar Omega, BMG, Ortenberg, Germany). Results were expressed as mmol Fe^2+^ per gram of hydrolysate (soluble fraction) in dry basis.

### 2.8. Determination of Anti-Inflammatory Activity

The murine macrophage cell line RAW 264.7 was maintained and cultivated as recommended by the American Type Culture Collection (Rockville, MD, USA). Cells were seeded at a density of 2 × 10^5^ cells per well in 24-well plates. Cells were pre-exposed for 1 h to a sample concentration equivalent to 0.5 mg hydrolysate soluble fraction per milliliter of growth media. Afterwards, cells were stimulated using 1 µg/mL of lipopolysaccharide (LPS) O55:B5 from *Escherichia coli* (Sigma-Aldrich, Madrid, Spain) for 23 h. Untreated cells were included as the negative control of inflammation and LPS-treated cells without sample extracts were the inflammatory model of reference. After 24 h, we collected supernatant for quantification of tumor necrosis factor (TNF)-α, monocyte chemoatractant protein (MCP)-1, and interleukin (IL)-6 by enzyme-linked immunosorbent assay kits (Diaclone, Besacon Cedex, France). The absorbance was read at 450 nm using a Synergy HT microplate reader (Biotek Instruments, VT, USA). Results were expressed as percentage inhibition relative to LPS-treated cells without sample extracts. Cell viability was determined in 96-well plates seeded at a density of 5 × 10^4^ cells per well using the Cell Titer 96 AQueous One Solution Proliferation Assay kit (Promega Biotech Ibérica, Madrid, Spain). Cell viability was expressed as percentage relative to untreated cells.

### 2.9. Modeling and Optimization of Hydrolytic Treatments

For response surface modeling, we built a mathematical model for each response fitting a second order polynomial function. WB solubilization yield (Ys), TSPC, FA, ORAC, ABTS, DPPH, FRAP, MCP-1, IL-6, and TNF-α were selected as response variables. Each response variable (Y) was described by the regression model shown in Equation 2:

(2) $$Y=\beta_{0}+\overset{n}{\underset{i=1}{\sum }}\beta_{i}X_{i}+\overset{n}{\underset{i=1}{\sum }}\beta_{ii}X_{i}^{2}+\overset{n}{\underset{i=1}{\sum }}\overset{n}{\underset{j=1}{\sum }}\beta_{ij}X_{i}X_{j}$$

where $\beta_{0}$ is a constant coefficient, and $\beta_{i}$_,_ $\beta_{ii}$, and $\beta_{ij}$ are the linear, quadratic, and interaction coefficient, respectively. $X_{i}$ and $X_{j}$ are the factors (temperature, pH, and time) and *n* is the number of response variables studied. The significance of the coefficients was investigated through one-way analysis of variance (ANOVA) using the Statistica v.7.0 software.

An ANOVA test was applied to determine the significance of the second order model. The latter was considered satisfactory when the regression was significant, and a non-significant lack of fit was obtained for the selected confidence level (α = 0.05). Moreover, the coefficient of determination (*R*^2^) and the adjusted coefficient of determination (*R*^2^*-adj*) were evaluated to confirm that variation of the data was mainly explained by the model. Three-dimensional response surface plots were obtained to illustrate the effects of the independent factors on the response variables. Multiresponse optimization was performed using the Desirability Function in Statistica v.7.0. The following desirability criteria were selected: maximum concentration of soluble phenolic compounds, and antioxidant and anti-inflammatory properties in WB hydrolysates.

### 2.10. Statistical Analysis

Data were expressed as the mean ± standard deviation. ANOVA and post hoc Duncan’s test was used to identify differences between mean values. Multivariate analysis of variance (MANOVA) test was used to identify significant effects of hydrolysis factors (pH, temperature, and time) on response variables. Pearson correlation coefficients and principal component analysis (PCA) were performed on centered and standardized data to elucidate the relationships among variables of the phenolic profile and bioactivity of samples. 

## 3. Results

### 3.1. Ultraflo XL Showed Higher Efficiency for the Release of Bound Phenolic Acids from WB

First, the study focused on the selection of the most efficient enzyme for the solubilization of bound phenolic acids from WB. To that purpose, we characterized 13 commercial enzymes containing a mixture of glycosidase activities (Table S1) in terms of enzymatic profile and their ability to disintegrate WB matrix on the basis of the release of monosaccharides and FA derivatives. Enzymatic profile of commercial enzymes is shown in Table S2. CELase activity ranged between not detected and 23.98 U/g. The highest values for CELase activity were found by Depol 40L, Depol 686L, Depol 740L, Depol 793L, Shearzyme, Ultraflo XL, and Viscoferm. Similarly, commercial enzymes showed a wide variation for XYLase activity ranging between 1.84 and 21.93 U/g. Depol 686L showed the highest XYLase activity while Viscozyme showed the lowest activity. Regarding AFase activity, we found higher values (3.07–3.41 U/g) of Depol 670L, Pectinase 62L, and Viscozyme compared with the other enzymes tested, whereas Depol 761L and 333MDP showed 100-fold lower AFase activity (0.01 and 0.03 U/g, respectively). Finally, Depol 670L, Depol 686L, Depol 740L, and Ultraflo XL were the only preparations that exhibited FEase activity toward *p*-nitrophenyl esters of FA. Depol 740L expressed the highest levels of FEase activity (290.45 mU/g) followed by Ultraflo XL (175.39 mU/g).

Monosaccharide and WB solubilization yields were studied after WB enzymatic treatment (Table 1) to compare the ability of the different multi-enzymatic preparations to disintegrate WB polysaccharides (cellulose, β-glucan, AX, and arabinogalactans). Increased monosaccharide yields were observed after enzymatic hydrolysis, although a variation in carbohydrates composition was found among treatments. Viscozyme was the most efficient enzyme, releasing glucose monomers from WB polysaccharides (4.43-fold increase), while Viscoferm and Shearzyme gave rise to the highest xylose yields (74.7- and 83.0-fold, respectively). Despite all enzymatic treatments showing AFase activity, only seven enzymatic treatments significantly increased arabinose content between 5.84- and 34.5-fold compared to the control, with Ultraflo XL and Depol 740L being the most efficient enzymes in terms of releasing arabinose residues from WB polysaccharides. Regarding galactose, only Depol 40L, Pectinase 62L, and Viscozyme treatments were capable of increasing yields of this monosaccharide, with Viscozyme being the enzyme with the highest efficiency.

WB solubilization yield showed a significant variation among enzymatic treatments (Table 1). Ultraflo XL followed by Viscozyme and Viscoferm produced higher solubilization yields of 79%, 69%, and 68% by weight, respectively. This parameter was positively correlated (*r =* 0.79, *p* = 0.001) with monosaccharide yield (see Figure S1), suggesting that WB solubilization was directly related to enzymatic breakdown and consequently release of WB polysaccharides.

The efficiency of the 13 enzymatic treatments to release bound phenolic compounds from WB is shown in Table 2. Except in the cases of Depol 333MDP, Pectinase 62L, and Viscozyme, we found that TSPC increased between 1.5- and 2-fold in WB as a consequence of enzymatic treatment. The highest TSPC yields were achieved using Ultraflo XL (5.49 mg ferulic acid equivalents (FAE)/g bran). Free FA and derivatives were tentatively identified based on accurate mass measurements by high-performance liquid chromatography coupled to electrospray ionization and quadrupole time-of-flight mass spectrometry (HPLC-ESI-QTOF-MS) and literature comparison. A total of five compounds were identified in WB samples (Table S3). FA and vanillin ferulic acid (VFA) were detected in untreated WB (control), whereas dihydroferulic acids isomers (DFA i1 and i2) and decarboxylated forms of DFA (DFA dc) were only detected in WB after the application of enzymatic treatments (data not shown).

A wide variation in the solubilization efficiency of FA derivatives was observed among enzymatic treatments (Table 2). Amounts of free FA and derivatives were largely obtained using Depol 670L, Depol 686L, Depol 740L, and Ultraflo XL treatments, which was in consistency with the FEase activity exhibited by these commercial preparations. Among these glycosidases, Ultraflo XL showed the highest yields of free FA (799.5 μg/g of bran), DFA i1 (555.5 μg/g of bran), DFA i2 (586.5 μg/g of bran), DFA dc (124.4 μg/g of bran), and VFA (233.9 μg/g of bran). Moreover, Ultraflo XL was the only enzymatic treatment able to release DFA i1 from WB matrix.

**Figure 1:**
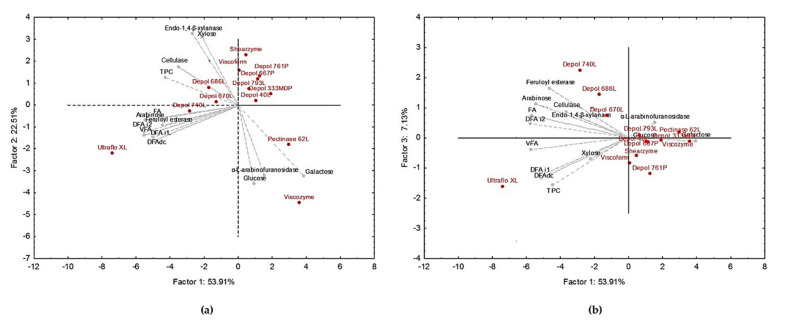
Bi-plots of principal component analysis (PCA): Factor 1 vs. Factor 2 (**a**) and Factor 1 vs. Factor 3 (**b**) separates enzymatic treatments in terms of enzymatic activity profile, phenolic acids, and monosaccharide composition of WB hydrolysates. Abbreviations: decarboxylated form of dihydroferulic acid (DFA dc); dihydroferulic acid isomer 1 (DFA i1); dihydroferulic acid isomer 2 (DFA i2); ferulic acid (FA); total phenolic compounds (TPC); vanillin ferulic acid (VFA).

Principal component analysis (PCA) was used to the variation in monosaccharide and phenolic composition of WB hydrolysates treated by the different commercial glycosidases and to identify correlations among variables (Figure 1). The first, second, and third factors explained 83.6% of the total variance. All the samples were well separated on the plane. Points represent enzymatic treatments, while the vectors are related to variables (enzymatic activities, monosaccharides, and phenolic acids). FEase, phenolic acids, as well as arabinose were found at the negative end of Factor 1, and therefore these variables were well explained by this principal component and correlated with each other (Figure 1a). Factor 2 described the variation in XYLase, CELase, and xylose (located in the positive branch), as well as the variation in AFase, glucose, and galactose (found at the negative end). Factor 3 explained mainly variation in TSPC (Figure 1b). The PCA allowed a clear separation of WB enzymatic treatments. Differences between enzymatic treatments mainly extended along the first principal component. This meant that treatments mainly varied regarding FEase activity, phenolic acids, and arabinose content. Most of enzymatic treatments were clustered in the right part of the bi-plots (Figure 1a,b); thus, WB hydrolysates obtained by Depol 761P, Depol 667P, Depol 793L, Depol 333MDP, Depol 40L, Viscoferm, and Shearzyme were characterized by lower amounts of TSPC, phenolic acids, and arabinose. These characteristics were also applicable for Pectinase 62L and Viscozyme treatments, which, additionally, were defined by high AFase activity, galactose, and glucose and low XYLase activity and xylose content. Depol 686L, Depol 670L, and Depol 740L treatments containing FEase activity generated hydrolysates with an intermediate content of TSPC, ferulic derivatives, and arabinose. Finally, Ultraflo XL was separated in the left bottom side of bi-plots (Figure 1a,b) due to its high content in TSPC, ferulic derivatives, and arabinose.

Multiple regression analysis was performed to identify associations between variables (Table S4). Regression analysis confirmed positive correlations between arabinose content and FEase (*r* = 0.92, *p* ≤ 0.001). A direct relationship was also observed between arabinose and FA derivatives (*r* = 0.64-0.91, *p* ≤ 0.05) (Table S4). FA content was positively associated with the yields of identified DFA derivatives and VFA (*r* = 0.73-0.95, *p* ≤ 0.01), CELase (*r* = 0.59, *p* ≤ 0.05), and FEase activities (*r* = 0.76, *p* ≤ 0.01).

According to the above results, we selected Ultraflo XL and used it for further experiments. In addition, we selected TSPC and FA as response variables for further optimization of the enzymatic process as both parameters were strongly associated with the content of DFAs and VFA (*r* = 0.62-0.95, *p* ≤ 0.05).

### 3.2. Autoclave and Subsequent Ultraflo XL Treatment of WB Exerted Synergistic Effects on Solubilization of Phenolic Compounds

Solubilization yield of phenolic compounds in WB was further optimized by assessing the effect of autoclave treatment on TSPC and free FA of WB before and after Ultraflo XL addition (Table 3). Autoclave treatment enhanced TSPC and free FA content in WB (2.4- and 41-fold increase compared to control, respectively, *p* ≤ 0.05) although the combined action of autoclave and Ultraflo XL treatments showed a synergistic action increasing even more TSPC and free FA contents by 4.5- and 329.5-fold, respectively. Therefore, subsequent optimization experiments were performed including autoclaving as pretreatment before enzymatic hydrolysis of WB.

### 3.3. Hydrolysis Conditions Explained the Variation in the Solubilization Yield, Free Phenolic Compounds, and Antioxidant and Anti-Inflammatory Activities of WB Hydrolysates

RSM was employed to optimize operating conditions of enzymatic treatment of WB. Incubation temperatures and solvent pH values ranging from 47 to 63 °C and 3.32 to 6.7, respectively, were selected taking into consideration the declared working temperature and pH range of commercial enzymes (Table S1). Time ranging from 0.5 to 27.5 h was selected based on the kinetic study of the release of free FA from WB by Ultraflo XL (Figure S2). In this single factor experiment, we observed that FA content increased up to 24 h, and therefore this value was set up as the maximum level in the CCD. The face-centered design setting of factors (temperature, pH, and time) and experimental values of response variables is shown in Table 4.

Reaction conditions applied during WB hydrolysis resulted in a significant variation in the solubilization yield, free FA content, and antioxidant and anti-inflammatory properties of WB hydrolysates (Table 4). MANOVA was performed to know the contribution of temperature, pH, and time to the variability of each response variable. MANOVA indicated that pH (*p* ≤ 0.001) and secondly time (*p* ≤ 0.01) were the main factors contributing to the variation of WB solubilization yield, although temperature did not exert a significant effect. The highest WB solubilization yield was observed when enzymatic treatment of WB was performed at pH ≥ 6 over 14 h (78.6–84.7%), regardless of the temperature used. Similarly, pH was the main factor contributing to the variation of TSPC and FA in WB hydrolysates, followed by time and temperature (*p* ≤ 0.001). The highest yield of TSPC was obtained at acidic pH between 4 and 5 at 50-55 °C for 22-27.5 h, while maximum free FA content was reached at 50 °C, pH 6, and times between 6 and 22 h.

For a complete evaluation of the total antioxidant activity of WB hydrolysates, we performed several assays: ORAC (based on hydrogen atom transfer mechanism), ABTS and DPPH (based on single electron transfer mechanism), and FRAP (based on chelation of transition metals). MANOVA results indicated that pH was the main factor that contributed to overall variation in the antioxidant activity of WB hydrolysates, as was determined by the four antiradical methods (*p* ≤ 0.001). The second factor contributing to the variation observed in the antiradical activity of WB hydrolysates was temperature for ABTS and DPPH assays and time for ORAC and FRAP assays. WB hydrolysates produced at 50 °C, pH 4, for 22 h showed the highest antioxidant activity regardless of the in vitro assay used.

Anti-inflammatory activity of WB hydrolysates produced by Ultraflo XL was studied using LPS-induced RAW264.7 macrophages. Firstly, cytotoxicity assays were performed to confirm the safety of cell treatments. Non-significant (*p* ≤ 0.05) differences in cell viability between treated (0.5 mg/mL) and non-treated cells confirmed that WB hydrolysates did not exert a cytotoxic effect (data not shown). Differences in the ability of hydrolysates to inhibit pro-inflammatory cytokines were observed among experiments (Table 4). Time was the main factor influencing the ability of WB hydrolysates to reduce MCP-1 and TNF-α secretion (MANOVA, *p* ≤ 0.001), while temperature was the factor that exclusively explaining the observed variability in IL-6 inhibition of WB hydrolysates (MANOVA, *p* ≤ 0.001). The hydrolysates produced at pH 5 for 14 h exhibited the greatest inhibition of TNF-α (57.7–63.5%) and MCP-1 levels (48.9–52.0%), regardless of the temperature used for WB hydrolysis (ANOVA, *p* <0.05). In the case of IL-6, WB hydrolysates with the highest inhibitory activity (54.9–60.1%) were obtained at 50–60 °C, pH = 4-6, for 22 h.

### 3.4. Statistical Optimization of Temperature, pH, and Time during WB Enzymatic Hydrolysis

Models were built to describe the effects of factors on response variables. Table 5 collects mathematical models with best fitting results (*p*-values ≤ 0.05 and non-significant lack of fit) and those that explained more than 75% of the variability of experimental data (*R*^2^ and *R*^2^-*adj* ≥ 0.75) [23]. Only the regression coefficients with significant probability levels (*p* < 0.05) were included in the predictive models. TSPC, FA, ORAC, DPPH, FRAP, and inhibition of MCP-1 followed a second polynomial model whereas regression models generated for solubilization yield, TNF-α, IL-6, and ABTS were not accepted (*R*^2^ < 0.75) and not included for multi-response optimization. The relationship between the two factors with higher contribution on response variables is represented in Figure S3. In 3D surface plots, the factor with lower contribution on the response was fixed at the optimum level.

All response variables were affected by linear, quadratic, and interaction effects of temperature, pH, and time. 3D surface contour plots (Figure S3) indicated that phenolic compounds and antioxidant activity in WB hydrolysates showed a decreasing trend with increasing temperature levels (Figure S3b,d). The pH exerted dominant linear and quadratic effects in most of response variables included in Table 5, with the exception of MCP-1. An increase trend with increasing pH levels up to 5–6 was observed for TSPC, FA, and biological activities of WB hydrolysates. pH values above 6 gave rise a reduction of these parameters. Regarding time, better extraction of TSPC and FA, as well as higher antioxidant potential was observed in WB hydrolysates at longer times of incubation (Table 3, Figure S3). A similar trend was observed for the ability of hydrolysates to inhibit MCP-1, although incubation times over 20 h resulted in a partial loss of this biological activity.

Desirability (*D*) was used to find the optimal processing conditions for production of hydrolysates with maximized yields of free phenolic acids and bioactivity. This analysis generated desirability plots where desirability values approaching to 1.0 were most preferable for concurrent improvement of soluble phenolics yield, as well as antioxidant and anti-inflammatory activities (Figure S4). The hydrolysis parameters providing the highest desirability value (*D* = 0.74) to maximize the nutraceutical value of WB were 47 °C, pH = 4.4, and 20.8 h (Table 6). Under these optimal conditions, we predicted the soluble fraction of WB hydrolysates to contain 664.8 mg FAE/100 g of TSPC and 222.3 µg/100 g of FA (Table 6). WB hydrolysates were predicted to exert an antioxidant activity of 21.5 g TE, 6.1 g TE, and 0.9 mmol Fe^2^ per 100 g of hydrolysate as determined by ORAC, DPPH, and FRAP assays, respectively. Finally, 0.5 mg of WB hydrolysates were predicted to reduce 35.1% MCP-1 levels in LPS-stimulated macrophages. Validation of desirability model was performed by comparing experimental data obtained for a combination of factors in the optimal desirability region defined by temperatures between 47 and 56 °C, pH between 4 and 5.5, and times between 14 and 22 h (Figure S4). As shown in Table 6, experimental values of all responses obtained at 50 °C, pH 5, and 22 h were found to be in the range of predicted interval (95% confidence) given by the software. These results verified the validity of the optimization model. As compared to non-hydrolyzed WB (control), hydrolysates produced under optimal reaction conditions were predicted to provide 2.2-fold and 4.3-fold of TSPC and free FA, respectively. Regarding bioactivity, hydrolysates were predicted to exhibit 1.5-, 2.8-, and 2.0-fold higher potential to scavenge oxygen radicals, chelate transition metals, and inhibit MCP-1 production in activated macrophages, respectively.

## 4. Discussion

After maize, wheat is the second most produced cereal crop worldwide, with 734 million tons produced annually, of which about 20% are converted to bran (150 million tons/year) [24]. Minor amounts of WB are applied in food development, despite this cereal milling by-product being a source of valuable biomolecules that can be converted in value-added products through multiple valorization routes [25]. In this context, the objective of this study was to enhance the WB biofunctionality towards antioxidant and anti-inflammatory properties through the solubilization of phenolic acids by a combination of autoclave and enzymatic treatments. Enzymatic treatments have been pointed out as environmentally friendly, energy efficient, and selective biorefinery processes with a great potential to provide from feedstock tailored products in terms of chemical composition, and technological and bioactive features [26].

A number of enzymes are required to completely degrade the heterogeneous network of WB polysaccharides [27]. Synergism between CELase, XYLase, *β*-xylosidase, AFase, FEase, and *α*-D-glucuronosidases is vital for efficient polysaccharide backbone degradation and production of target nutraceutical compounds. Therefore, we proposed an approach of enzyme selection to improve the economic viability of production of value-added functional ingredients. Thirteen enzyme cocktails including a wide portfolio of cellulosic and hemicellulosic activities were characterized and compared for their specific activity and ability to disintegrate WB polysaccharides and release of phenolic compounds. Soluble fraction of WB accounted for 46% of its dry weight. These soluble fractions could be composed of soluble fiber components such as β-glucans and native oligosaccharides, as well as free monosaccharides, amino acids, starch, proteins, minerals, vitamins, and soluble phytochemicals. After enzymatic treatment of WB, monosaccharides such as glucose, xylose, and arabinose increased significantly (Table 1), which indicated that all enzymatic preparations were able to disintegrate AX and cellulose due to their CELase, XYLase, and AFase activities (Table S2). The sum of monosaccharides increased greatly with the different treatments as well as the solubilization yield, although not in the same proportion. Monosaccharide content was positively correlated with solubilization yield, although release of other compounds such as arabinoxilooligosaccharides, amino acids, and peptides could also contribute to the increase in the solubilization yield. A significant variation in the monosaccharide release and WB solubilization among treatments was observed (Table 1), probably due to the distinct specific activity among enzymatic preparations. Ultraflo XL, Viscoferm, and Viscozyme treatments produced higher monosaccharide yields (87–101 mg/g bran), which in total was equivalent to solubilization of 68–79% by weight of total dry matter (Table 1). These results indicated that these three enzymes were more efficient to disintegrate and solubilize WB components.

Regarding phenolic composition, TSPC and free FA contents of WB (Table 2) were in the range reported for brans from *Triticum* spp. (0.5-2 mg GAE/g of bran and 2.9-9.5 μg/g of bran, respectively) [28,29]. Most enzyme treatments used in the present study significantly increased TSPC and individual content of FA derivatives in WB (Table 2). Enzyme cocktails containing FEase (Depol 670L, Depol 686L, Depol 740L, and Ultraflo XL) were the most efficient enzymes releasing FA derivatives producing WB hydrolysates composed predominantly by FA monomers and lower amounts of DFA and VFA. The presence of DFA in WB hydrolysates could be only attributed to the presence of FEase of type A, which is capable of hydrolyzing the main DFA isomers of WB (5-5′ and 8-*O*-4′ DFAs) [12]. Depol 40L, Depol 793L, Shearzyme, and Viscoferm showed lower FA yields despite FEase activity not being detected in these enzymatic preparations (Table S2). A possible explanation to this observation could be to the lack of affinity of these enzymes for the synthetic substrate used in the determination of FEase activity.

From the PCA analysis it was confirmed that Depol 686L, Depol 670L, and Depol 740L treatments produced hydrolysates with an intermediate content of TSPC and FA derivatives while Ultraflo XL produced hydrolysates with the highest content of free phenolic acids (Figure 1). Similar results were found by Kim et al. [30], who pointed out that Ultraflo XL, Celluclast, and Pentopan were more effective in increasing the amount of free phenolic acids from rice bran. The high WB solubilization yield (almost 80%) and release of monosaccharides and phenolic acids produced by Ultraflo XL determined the selection of this enzyme for further optimization experiments.

Multiple regression (Table S4) and PCA analysis (Figure 1) provided some relationships that explained the higher efficiency of Ultraflo XL compared to the other enzymatic treatments screened. The significant positive correlation between individual content of FA derivatives, CELase, FEase, and arabinose indicated that the combined action of CELase, AFase, and FEase was determinant in terms of enhancing the release of phenolic acids, with results being in agreement with previous studies [31]. The highest efficiency of Ultraflo XL to release arabinose residues from WB suggested a higher AFase activity of this enzyme compared to other enzymatic preparations. This hypothesis could not be corroborated with the determination of AFase activity, probably due to a lower substrate affinity of Ultraflo XL to *p*-nitrophenyl arabinofuranoside. The fact that Ultraflo XL contains the highest AFase and high FEase, XYLase, and CELase activities acting cooperatively explained its higher efficiency to disintegrate and solubilize polysaccharides and phenolic acids from WB. These results are in agreement with previous studies describing Ultraflo XL as an enzymatic preparation from *Humicola insolens* that contained a balance of glycosidases and esterases capable of solubilizing efficiently biomass of agroindustrial coproducts by over 50% [32]. Specifically, Ultraflo XL was reported to contain cellulase and xylanase activities, although it may lack a specific family 10 xylanase and enzymes capable of disrupting microcrystalline cellulose. Finally, FEase of Ultraflo XL have shown the ability to release FA and 5-5′DFA and a lower proportion of 8-*O*-4′ DFA from WB in agreement with our results.

In order to enhance the susceptibility of Ultraflo XL to disintegrate the complex network of heterogeneous polysaccharides of WB and release phenolic acids, we applied autoclave treatment as a pretreatment before enzyme addition (Table 3). Hydrothermal treatments including autohydrolysis (80 °C for 10 min), boiling (100 °C, 20 min), autoclave (120 °C, 1 bar, 20 min), and steam explosion (165–200 °C, 7-16 bar, 0.75–5 min) have been explored for WB valorization in terms of FA release due to their depolymerization effects and solubilization of hemicellulosic and cellulosic polysaccharides [25]. As compared to reported hydrothermal WB pretreatments, a higher relative fold increase was found for TSPC and FA in the present study after autoclave treatment (121 °C, 1 bar, 30 min) of WB [15,33]. Thermal hydrolysis of ester linkages between phenolic acids and polysaccharides was reported by Gong et al. [34] in barley bran, which could explain the increased amounts of phenolic acids in WB after autoclave processing. Application of higher temperatures of up to 130 °C during autoclave treatment of WB have also been reported as beneficial in terms of increasing storage stability, antioxidant properties, free ferulic acid, and apigenin-6-C-arabinoside-8-C-hexoside [35]. Combination of autoclave and Ultraflo XL treatments produced a further increase of TSPC and FA yields. These results were similar to those found by Jiang et al. [36], showing maximum FA yields after hydrothermal treatment of corn bran at 165 °C for 40 min and subsequent hydrolysis by an equal mixture from *Aspergillus oryzae* and *Eupenicillium parvum* crude protein extracts. Increased hydrolytic yield observed by combination of hydrothermal/pressure and enzymatic treatments is based on an initial heat/pressure-induced unspecific breakdown of hemicellulosic and cellulosic polysaccharides and feruloyl ester linkages that favors the subsequent exposure of cell components to enzymatic digestion [15].

The relationships of the specific variation of total solubilization yield (Ys), TSPC, and FA content as affected by reaction conditions during Ultraflo XL treatment of WB were also studied (Table 4). Identification of processing parameters influencing the yield of soluble phenolic compounds was useful to improve WB functional value through their optimization. Solvent pH was the main factor affecting WB biomass solubilization, TSPC, and FA content in the enzymatic process. Time and temperature showed lower effects on the solubilization of WB components, with the exception of temperature, which did not significantly influence WB solubilization yield. Predictive models of experimental data indicated that TSPC and free FA were influenced by linear, quadratic, and interaction effects of temperature, pH, and time in hydrolytic treatments of WB (Table 5). There are examples of these behaviors in the literature. Recently, Lau et al. [37] documented linear effects of temperature and interaction effects of pH and XYLase concentration as factors influencing the enzymatic-assisted extraction of FA from sweetcorn cob. In this study, FA increased with increasing pH values (from pH = 4 to pH = 6) and temperatures (from 20 to 50 °C), after which started to decrease. Optimum pH values and temperatures for the hydrolytic activity of FEase from *H. insolens* have reported to be between pH = 5 and 6, and 50 and 65 °C; however, higher pH and temperatures resulted in quick loss of activity [32,38]. This may in part explain the variation of free FA yields obtained after Ultraflo XL treatment as a function of pH and temperature. Regarding time, longer enzymatic treatments of WB resulted in higher TSPC and FA yields in accordance with previous studies [15,39].

WB antioxidant activity as determined by the four in vitro methods was within the range of previous studies [29,40]. Ultraflo XL increased the antioxidant activity of WB hydrolysates (Table 4) in consistency with previous works showing increased antioxidant activity after treatment of rice and wheat bran with different glycosidases [30,41]. The effects of glycosidases on antioxidant activity of WB could be partially attributed to the increased extractability of phenolic acids [30,41] generally reported to be potent radical scavengers and ferrous ion chelators. This was confirmed by significant positive correlations between TSPC and antioxidant activity evaluated using ORAC, DPPH, ABTS, and FRAP assays (Table S5). In contrast, FA content in hydrolysates showed no correlation with antioxidant activity, suggesting that other enzyme products were contributing to the antioxidant activity of hydrolysates. Feruloylated arabinoxylo-oligosaccharides also released from WB by glycosidases could be a good example due to their recently proven reducing activity and ability to scavenge DPPH, superoxide and hydroxyl radicals as well as to chelate metal ions [42]. 

It is important to mention that solvent pH, temperature, time, and their interaction (temperature–pH and pH–time) significantly affected the antioxidant activity of WB hydrolysates (Table 4). pH showed a higher effect than temperature and time in the antioxidant activity of WB hydrolysates. The impact of these factors on antioxidant activity could be explained by their effect on the enzymatic-assisted release of phenolic compounds and oligosaccharides. An increasing tendency in the antioxidant activity of hydrolysates was observed with increasing pH (up to pH = 5) and longer incubation times (15–27 h). This may be due to higher extraction of phenolic acids from WB under these conditions. Moreover, free radical scavenging activity of phenolic acids has shown a dependence on the magnitude of the dissociation of hydroxyl groups [43]. In acidic conditions (pH = 4) phenolic acids or esters weakly inhibited peroxidation, whereas with increasing pH, their antioxidant activity increased substantially being similar or higher than that of Trolox.

Although the main phenolic acids in WB, monomeric and dimeric FAs, are known to exhibit anti-inflammatory compounds at low concentrations [44], these compounds must be bioavailable to exert these beneficial effects. The fact that 95–98% of phenolic acids in WB are bound to cell walls, it was not surprising to observe that the treatment of LPS-stimulated macrophages with the soluble fraction of unhydrolyzed WB (0.5 mg/mL) showed a low anti-inflammatory activity: 17.3% inhibition of MCP-1 (Table 6), while inhibitory effects on TNF-α and IL-6 were not detected. In contrast, enzymatic treatment with Ultraflo XL significantly improved the anti-inflammatory activity of WB, which could be explained by the increased yields of soluble phenolic compounds. Other studies support this statement, reporting that wheat products with increased soluble/bound ratio of phenolic acids were associated with a lower pro-inflammatory response of LPS-induced blood cells [13]. In the present study, anti-inflammatory activity shared no direct relationship with TSPC and FA content of hydrolysates, suggesting the presence of other compounds with synergistic effects to impact on this trait. This is supported by the study of Mateo-Anson et al. [13] in which FA concentration did not explain the full anti-inflammatory effect observed for bioaccesible fractions of wheat aleurone submitted to a dynamic gastrointestinal digestion. Unlike antioxidant activity, temperature and time showed a higher effect on anti-inflammatory activity of hydrolysates, whereas pH did not affect (IL-6, TNF-α) or affected slightly (MCP-1) this biological function compared to the other two factors. These results suggest that the observed variation in the anti-inflammatory activity of hydrolysates is due to diversity of compounds, with yields mainly influenced by incubation temperature and time. FA, DFA, and feruloylated arabinoxylo-oligosaccharides with reported anti-inflammatory activity [44,45,46] have shown a time- and temperature-dependent release during WB hydrolysis by glycosidases [32].

Finally, operating conditions were optimized to maximize the nutraceutical value of WB hydrolysates. As compared to the control, under optimized conditions (47 °C, pH = 4.4, for 20.8 h) hydrolysates WB showed 4.2, 1.5, 2.9, and 2.0 times higher content of FA and bioactivities to scavenge oxygen radicals, chelate transition metals, and inhibit monocyte chemoattractant protein-1 secretion in macrophages, respectively. In the present study, WB solubilization and FA yields reached a maximum of 83% by weight and 222 mg/100 g of soluble fraction (equivalent to 5.1 g/kg of bran), values notably higher than those reported in the literature [16,26,47]. Pressurized liquid extraction of WB (1140 μm particle size) using pure water at 160 °C for 74 min has shown similar efficiency (0.38 g/kg WB) compared with alkaline hydrolysis using 2 M NaOH at 30 °C for 2 h treatment of WB solutions for FA recovery (0.4 g FA/kg of WB). Enzymatic hydrolysis of defatted WB with *Thermobacillus xylaniticus* cocktail at 50 °C, pH = 7.5, for 24 h allowed the release of 1.9 g/kg of bran [16]. More recently, an optimized process including three steps—rehydration of bran through steam explosion, protein and starch hydrolysis with Alcalase and Thermamyl, and a final enzymatic treatment with Pentopan and recombinant FEase from rumen microorganism)—yielded a maximum FA concentration of 1.05 g/kg bran [15]. Additionally, the process used in the present study required few steps, utilized only one enzyme, and did not involve the use of acid/alkali or toxic compounds.

## 5. Conclusions

The study demonstrates a novel approach where specific enzymatic treatment combined with WB hydrothermal pretreatment and optimization of pH, temperature, and time significantly improved the antioxidant and anti-inflammatory capacity of WB. Among 13 commercial glycosidases, Ultraflo XL was the most efficient enzyme in terms of solubilizing WB biomass, hemicellulosic and cellulosic polysaccharides in particular, while increasing FA and DFA bioaccesibility. The outstanding performance of Ultraflo XL was related to its balance consortium of enzymatic activities (CELase, XYLase, AFase, and FEase). Hydrolytic efficiency of Ultraflo XL in terms of phenolic acid yields could be improved with the application of autoclave. Hydrolysis conditions influenced the biological activity of hydrolysates, although free phenolic acid yields were not the only determinant in the observed variation. Adjusting solvent pH, incubation temperature, and time to pH = 4.4, 47 °C, and 20.8 h, respectively, maximized the bioactivity of WB hydrolysates. Results from this research provide an excellent opportunity for valorization of WB into valuable food ingredients. Further research studies are being performed to scale up the production and stabilization of WB hydrolysates and to validate their functional properties in vivo.

## Figures and Tables

**Table 1:** Monosaccharide content (mg/g of bran) and solubilization yield (Ys, %) of wheat bran (WB) treated by 13 commercial food-grade glycosidases.

Enzyme	Glucose	Xylose	Arabinose	Galactose	SUM	Y_S_
Control	20.36 ± 6.21 ^e^	0.38 ± 0.20 ^j^	0.33 ± 0.04 ^e^	2.27 ± 0.14 ^def^	23.34	51.32
Depol 333MDP	44.62 ± 13.07 ^bcd^	12.61 ± 1.19 ^gh^	1.11 ± 0.17 ^de^	2.89 ± 0.63 ^bcd^	61.23	55.10
Depol 40L	58.67 ± 15.28 ^b^	15.35 ± 2.98 ^efg^	2.17 ± 0.77 ^d^	3.22 ± 0.55 ^bc^	79.41	64.59
Depol 667P	46.01 ± 12.75 ^bcd^	11.37 ± 1.67 ^h^	1.05 ± 0.10 ^de^	2.40 ± 0.17 ^de^	60.83	61.07
Depol 670L	46.59 ± 9.46 ^bcd^	20.25 ± 3.34 ^c^	6.06 ± 0.96 ^bc^	2.03 ± 0.62 ^ef^	74.93	59.95
Depol 686L	46.55 ± 2.92 ^bcd^	17.55 ± 1.23 ^cde^	6.94 ± 1.96 ^b^	1.54 ± 0.46 ^f^	72.54	64.56
Depol 740L	52.58 ± 6.93 ^bcd^	16.06 ± 0.65 ^def^	11.38 ± 1.89 ^a^	2.28 ± 0.00 ^def^	82.30	62.54
Depol 761P	37.78 ± 2.43 ^d^	18.47 ± 2.47 ^cd^	1.20 ± 0.17 ^de^	2.45 ± 0.30 ^cde^	59.90	53.59
Depol 793L	48.94 ± 7.43 ^bcd^	13.36 ± 1.63 ^fgh^	1.17 ± 0.81 ^de^	2.45 ± 0.55 ^de^	65.92	57.43
Pectinase 62L	49.21 ± 3.83 ^bcd^	5.67 ± 0.94 ^i^	1.31 ± 0.73 ^de^	3.27 ± 0.29 ^b^	59.46	59.32
Shearzyme	41.07 ± 6.01 ^cd^	28.39 ± 2.89 ^b^	1.93 ± 1.03 ^d^	1.85 ± 0.13 ^ef^	73.24	65.88
Ultraflo XL	56.94 ± 18.31 ^bc^	17.49 ± 1.27 ^de^	11.05 ± 2.18 ^a^	1.78 ± 0.64 ^ef^	87.26	79.15
Viscoferm	58.76 ± 6.45 ^b^	31.53 ± 3.30 ^a^	4.86 ± 0.29 ^c^	1.88 ± 0.25 ^ef^	97.03	68.42
Viscozyme	90.33 ± 6.60 ^a^	4.28 ± 0.18 ^i^	1.28 ± 0.60 ^de^	5.06 ± 0.18 ^a^	100.95	68.88

Data are the mean ± standard deviation of two replicates. Different lowercase letters within a column are significantly different (*p* < 0.05, Duncan’s test). Hydrolysis conditions were enzyme to WB ratio of 1% (*w*/*w*), 40 °C, pH = 6, and 20 h. Ys: solubilization yield. SUM: indicates the total amount of monosaccharides.

**Table 2:** Contents of total soluble phenolic compounds (TSPC; mg ferulic acid equivalents/g of bran) and FA derivatives (µg/g of bran) in WB treated by 13 commercial food-grade glycosidases.

Enzyme	TSPC	FA	DFA i1	DFA i2	DFA dc	VFA
Control	2.80 ± 0.33 ^e^	11.64 ± 4.64 ^f^	nd	nd	nd	3.58 ± 1.03 ^c^
Depol 333MDP	3.30 ± 0.09 ^de^	53.40 ± 4.48 ^ef^	24.45 ± 2.16 ^b^	32.30 ± 0.15 ^d^	3.30 ± 0.82 ^d^	4.98 ± 5.81 ^c^
Depol 40L	4.36 ± 0.48 ^b^	120.10 ± 17.82 ^de^	19.18 ± 0.85 ^b^	39.75 ± 0.82 ^d^	8.63 ± 2.81 ^d^	20.64 ± 11.46 ^c^
Depol 667P	4.07 ± 0.13 ^bc^	43.51 ± 3.33 ^ef^	29.64 ± 2.75 ^b^	31.02 ± 0.03 ^d^	2.77 ± 0.75 ^d^	10.86 ± 1.72 ^c^
Depol 670L	4.38 ± 0.37 ^b^	528.93 ± 84.87 ^b^	60.27 ± 3.56 ^b^	340.92 ± 28.40 ^c^	73.96 ± 19.76 ^bc^	63.66 ± 6.46 ^b^
Depol 686L	3.61 ± 0.09 ^cd^	497.17 ± 67.03 ^b^	60.77 ± 3.51 ^b^	419.88 ± 0.27 ^bc^	85.35 ± 13.46 ^bc^	76.87 ± 3.73 ^b^
Depol 740L	4.14 ± 0.45 ^bc^	498.19 ± 82.85 ^b^	80.51 ± 5.15 ^b^	498.87 ± 1.65 ^b^	101.56 ± 12.66 ^b^	75.53 ± 29.96 ^b^
Depol 761P	4.52 ± 0.09 ^b^	49.97 ± 1.59 ^ef^	30.44 ± 0.04 ^b^	31.56 ± 4.43 ^d^	4.24 ± 1.66 ^d^	18.27 ± 13.98 ^c^
Depol 793L	4.27 ± 0.45 ^b^	240.56 ± 47.63 ^c^	25.41 ± 3.21 ^b^	42.57 ± 2.10 ^d^	15.81 ± 6.10 ^d^	11.35 ± 1.62 ^c^
Pectinase 62L	3.11 ± 0.10 ^de^	6.55 ± 1.79 ^f^	1.86 ± 0.39 ^b^	5.96 ± 0.06 ^d^	0.22 ± 0.31 ^d^	1.77 ± 0.49 ^c^
Shearzyme	4.33 ± 0.41 ^b^	111.43 ± 11.24 ^de^	28.00 ± 1.72 ^b^	38.42 ± 2.25 ^d^	15.72 ± 2.38 ^d^	8.20 ± 0.28 ^c^
Ultraflo XL	5.49 ± 0.64 ^a^	799.50 ± 34.19 ^a^	555.53 ± 16.12 ^a^	586.46 ± 13.06 ^a^	124.37 ± 87.85 ^a^	233.91 ± 13.96 ^a^
Viscoferm	4.65 ± 0.51 ^b^	179.75 ± 20.38 ^cd^	29.90 ± 2.73 ^b^	37.38 ± 2.58 ^d^	31.76 ± 10.27 ^cd^	13.34 ± 1.28 ^c^
Viscozyme	3.16 ± 0.14 ^de^	51.00 ± 5.35 ^ef^	12.89 ± 0.83 ^b^	31.57 ± 3.78 ^d^	3.99 ± 1.19 ^d^	8.19 ± 2.52 ^c^

Data are the mean ± standard deviation of two replicates. Different lowercase letters within a column are significantly different (*p* < 0.05, Duncan’s test). Abbreviations: decarboxylated form of dihydroferulic acid (DFA dc); dihydroferulic acid isomer 1 (DFA i1); dihydroferulic acid isomer 2 (DFA i2); ferulic acid (FA); total soluble phenolic compounds (TSPC); vanillin ferulic acid (VFA). Hydrolysis conditions were enzyme to WB ratio of 1% (w/w), 40 °C, pH 6, and 20 h.

**Table 3:** TSPC (mg ferulic acid equivalents/g of bran) and free FA (µg/g of bran) in WB after single or combined autoclave and Ultraflo XL enzymatic treatments.

Treatment	TSPC	FA
Control	2.80 ± 0.33 ^c^	11.64 ± 4.64 ^d^
Autoclave	6.85 ± 0.90 ^b^	478.24 ± 4.34 ^c^
Ultraflo XL	5.49 ± 0.64 ^b^	799.50 ± 34.19 ^b^
Autoclave + Ultraflo XL	12.61 ± 1.06 ^a^	3835.34 ± 119.78 ^a^

Data indicate mean ± standard deviation of two replicates. Different lowercase letters within a column are significantly different (*p* < 0.05, Duncan’s test). Abbreviations: ferulic acid (FA); total soluble phenolic compounds (TSPC). Autoclave treatment of WB (2 g/40 mL phosphate buffer at pH = 6) was performed at 121 °C, 1 bar for 30 min. Enzymatic hydrolysis of WB (2 g/40 mL phosphate buffer at pH 6) by Ultraflo XL (1%, *w*/*w*) was performed at 40 °C for 20 h.

**Table 4:** Solubilization yield (Ys, %), TSPC (mg ferulic acid equivalents/100 g hydrolysate [soluble fraction]), free ferulic acid (FA; mg/100 g hydrolysate [soluble fraction]), and antioxidant and anti-inflammatory activities of WB hydrolyzed by Ultraflo XL under different conditions of temperature (°C), pH, and time (h).

T	pH	t	Ys	TSPC	FA	ORAC^1^	ABTS^1^	DPPH^1^	FRAP^2^	MCP-1^3^	IL-6^3^	TNF-α^3^
55	3.32	14	42.9 ± 0.04 ^d^	564.6 ± 37.9 ^bc^	4.0 ± 0.7 ^h^	13.78 ± 2.01 ^de^	13.26 ± 1.08 ^cde^	4.21 ± 0.72 ^defg^	0.75 ± 0.11 ^fg^	28.55 ± 4.1 ^c^	2.74 ± 3.26 ^e^	50.6 ± 1.4 ^cd^
50	4	6	46.6 ± 10.2 ^bcd^	514.2 ± 39.4 ^cd^	77.1 ± 6.7 ^g^	16.99 ± 1.93 ^bcd^	14.61 ± 0.84 ^bcd^	6.09 ± 1.35 ^a^	0.85 ± 0.02 ^cd^	20.30 ± 7.7 ^d^	35.9 ± 10.4 ^b^	52.8 ± 9.8 ^bcd^
60	4	6	45.7 ± 8.8 ^cd^	447.6 ± 42.8 ^def^	6.7 ± 0.7 ^h^	15.16 ± 2.01 ^bcde^	13.28 ± 0.82 ^cde^	4.31 ± 1.30 ^def^	0.77 ± 0.01 ^ef^	41.02 ± 6.0 ^b^	17.1 ± 2.80 ^cd^	45.3 ± 4.2 ^d^
50	4	22	64.8 ± 5.6 ^abcd^	703.3 ± 25.8 ^a^	125.8 ± 1.5 ^f^	22.67 ± 2.07 ^a^	17.04 ± 0.53 ^a^	5.90 ± 0.28 ^a^	0.94 ± 0.02 ^a^	36.13 ± 2.0 ^ef^	60.1 ± 5.77 ^a^	nd
60	4	22	65.4 ± 11.9 ^abcd^	588.6 ± 35.3 ^b^	7.1 ± 0.5 ^h^	17.99 ± 1.57 ^bc^	15.37 ± 2.52 ^abc^	5.00 ± 0.16 ^bcd^	0.87 ± 0.01 ^bc^	nd	10.9 ± 0.9 ^de^	nd
55	5	0.5	61.1 ± 15.0 ^abcd^	498.4 ± 34.6 ^cde^	88.6 ± 3.0 ^g^	13.72 ± 3.41 ^de^	11.26 ± 1.47 ^ef^	4.18 ± 0.45 ^defg^	0.74 ± 0.05 ^fg^	5.29 ± 9.1 ^fg^	22.7 ± 9.9 ^c^	0.3 ± 0.5 ^ef^
47	5	14	64.1 ± 19.4 ^abcd^	562.3 ± 16.4 ^bc^	287.1 ± 3.9 ^c^	14.44 ± 3.36 ^cde^	14.61 ± 1.42 ^bcd^	5.50 ± 0.55 ^ab^	0.85 ± 0.01 ^bcd^	52.03 ± 6.6 ^a^	20.9 ± 9.0 ^cd^	63.5 ± 5.8 ^a^
55	5	14	69.6 ± 10.1 ^abc^	524.2 ± 52.3 ^bc^	271.6 ± 10.0 ^d^	15.30 ± 3.27 ^bcde^	14.20 ± 1.30 ^bcd^	5.29 ± 0.35 ^abc^	0.85 ± 0.04 ^bcd^	51.67 ± 1.3 ^a^	39.1 ± 4.2 ^b^	60.9 ± 2.7 ^ab^
63	5	14	59.3 ± 7.0 ^abcd^	528.7 ± 42.0 ^bc^	141.0 ± 11.0 ^e^	15.09 ± 1.34 ^bcde^	11.69 ± 1.57 ^ef^	4.64 ± 0.33 ^cde^	0.81 ± 0.04 ^de^	48.93 ± 2.3 ^a^	35.3 ± 3.9 ^b^	57.7 ± 2.4 ^abc^
55	5	27.5	71.9 ± 7.0 ^ab^	696.1 ± 40.7 ^a^	284.2 ± 7.0 ^cd^	18.42 ± 6.17 ^b^	15.94 ± 1.69 ^ab^	5.72 ± 0.14 ^ab^	0.91 ± 0.04 ^ab^	0.4 ± 0.0 ^g^	nd	0.3 ± 0.0 ^f^
50	6	6	66.6 ± 10.2 ^abcd^	377.1 ± 30.0 ^gh^	325.6 ± 27.4 ^a^	11.79 ± 1.04 ^e^	10.92 ± 0.72 ^f^	3.55 ± 0.25 ^fgh^	0.66 ± 0.04 ^h^	29.6 ± 5.9 ^c^	39.5 ± 10.9 ^b^	48.7 ± 15.5 ^cd^
60	6	6	68.1 ± 13.0 ^abcd^	350.2 ± 18.8 ^h^	305.1 ± 7.4 ^b^	11.57 ± 0.64 ^e^	10.94 ± 0.64 ^f^	3.49 ± 0.24 ^fgh^	0.66 ± 0.01 ^h^	44.9 ± 5.5 ^ab^	18.2 ± 4.09 ^cd^	55.9 ± 1.0 ^abc^
50	6	22	84.7 ± 9.9 ^a^	432.1 ± 34.5 ^efg^	334.4 ± 3.1 ^a^	14.31 ± 2.60 ^cde^	11.59 ± 0.76 ^ef^	3.22 ± 0.17 ^h^	0.67 ± 0.03 ^h^	24.9 ± 3.9 ^cd^	56.4 ± 6.1 ^a^	6.5 ± 5.2 ^ef^
60	6	22	83.7 ± 9.9 ^a^	354.0 ± 28.9 ^h^	311.5 ± 10.5 ^b^	14.01 ± 3.26 ^de^	10.78 ± 1.01 ^f^	4.12 ± 1.08 ^efg^	0.66 ± 0.02 ^h^	17.4 ± 7.9 ^de^	54.9 ± 4.7 ^a^	10.6 ± 2.3 ^e^
55	6.7	14	78.6 ± 15.9 ^a^	387.2 ± 37.9 ^fgh^	305.2 ± 10.7 ^b^	13.48 ± 2.68 ^ed^	12.63 ± 2.27 ^def^	3.41 ± 0.16 ^gh^	0.69 ± 0.05 ^gh^	2.1 ± 4.1 ^g^	1.5 ± 0.3 ^e^	10.4 ± 4.2 ^e^
**MANOVA**												
T	MS		12.7	20,266 ***	29,010 ***	2.8 x 10^7^ ***	1.3 x 10^7^ ***	2.2 x 10^6^ **	0.009 **	2897.7 ***	3373.4 ***	2680.1 ***
pH	MS		1375.9 ***	164,046 ***	385,120 ***	2.2 x 10^8^ ***	7.1 x 10^7^ ***	1.6 x 10^7^ ***	0.199 ***	671.6 **	869.9	126.9
t	MS		700.1 **	58,439 ***	15,733 ***	1.1 x 10^8^ ***	1.2 x 10^7^ ***	9.6 x 10^5^ *	0.028 ***	5165.1 ***	472.9	9522.4 ***

Data are mean values ± standard deviation of three replicates. Different lowercase letters in the same column indicate significant differences for mean values (one-way ANOVA, Duncan′s test, *p* ≤ 0.05). Significance indicators: * *p* ≤ 0.05, ** *p* ≤ 0.01, and *** *p* ≤ 0.001 (MANOVA). Abbreviations: 2,2’-azino-bis(3-ethylbenzothiazoline-6-sulfonic acid) (ABTS); 2,2-diphenyl-1-picryl-hydrazyl-hydrate (DPPH); ferric reducing antioxidant power (FRAP); ferulic acid (FA); interleukin 6 (IL-6); mean of squares (MS); monocyte chemoattractant protein 1 (MCP-1); multivariate analysis of variance (MANOVA); not detected (nd); oxygen radical absorbance capacity (ORAC); temperature (T); time (t); total soluble phenolic compounds (TSPC); tumor necrosis factor α (TNF-α). ^1^ Expressed as milligram Trolox equivalents (TE) per 100 g of soluble fraction; ^2^ expressed as millimole Fe^2+^/ 100 g of soluble fraction; ^3^ expressed as percentage inhibition.

**Table 5:** Predicted models and regression coefficients for the response variables of WB hydrolysates.

Response Variable	Mathematical Models *	*R^2^*	*R^2^-adj*
TPSC	$Y=-5.7T+269.7\mathrm{pH}-29.2{\mathrm{pH}}^{2}+26.1t-3.9\mathrm{pHt}$	0.78	0.76
FA	$Y=-2632.9+65.7T-0.8T^{2}+316.9\mathrm{pH}-39.6{\mathrm{pH}}^{2}+27.1t-0.4t^{2}+3.6\mathrm{TpH}-0.2\mathrm{Tt}-0.5\mathrm{pHt}$	0.93	0.92
ORAC	$Y=45.9-1.1T-1.5{\mathrm{pH}}^{2}+0.02t^{2}+0.2\mathrm{TpH}-0.1\mathrm{pHt}$	0.80	0.79
DPPH	$Y=-0.01T^{2}-0.5{\mathrm{pH}}^{2}-0.003t^{2}+0.1\mathrm{TpH}+0.01\mathrm{Tt}-0.02\mathrm{pHt}$	0.81	0.79
FRAP	$Y=-2.2+0.01T-0.001T^{2}+0.33\mathrm{pH}-0.04{\mathrm{pH}}^{2}+0.03t-0.0003t^{2}-0.003\mathrm{pHt}$	0.79	0.77
MCP-1 (i%)	$Y=19.91-0.2{\mathrm{pH}}^{2}+4.5t-0.2t^{2}-0.02\mathrm{Tt}$	0.80	0.79

* Models significance *p* ≤ 0.05 and lack of fit *p* ≥ 0.05 (ANOVA). Abbreviations: 2,2-diphenyl-1-picryl-hydrazyl-hydrate (DPPH); ferric reducing antioxidant power (FRAP); ferulic acid (FA); inhibition percentage (i%); monocyte chemoattractant protein 1 (MCP-1); oxygen radical absorbance capacity (ORAC); temperature (T); time (t); total soluble phenolic compounds (TSPC).

**Table 6:** Combination of enzymatic hydrolysis factors and predicted values at optimum desirability (*D*) for phenolic content, and antioxidant and anti-inflammatory activity.

Optimum *D* Value	Factors at Optimum *D* Value	Response Variables	Predicted Values	Experimental Values	Control
0.74	47 °C, pH = 4.4, 20.8 h	TSPC (mg FAE/100g)	664.80	703.3 ± 25.8	301.52 ± 16.11
		FA (mg/100 g)	222.27	125.8 ± 1.5	52.66 ± 1.58
		ORAC (g TE/100 g)	21.5	22.67 ± 2.07	14.65 ± 2.48
		DPPH (g TE/100 g)	6.10	5.90 ± 0.28	6.37 ± 0.40
		FRAP (mmol Fe^2+^/100 g)	0.91	0.94 ± 0.02	0.32 ± 0.01
		MCP-1 (i%)	35.09	36.1 ± 2.0	17.34 ± 2.32

Abbreviations: 2,2-diphenyl-1-picryl-hydrazyl-hydrate (DPPH); ferric reducing antioxidant power (FRAP); ferulic acid (FA); inhibition percentage (i%); monocyte chemoattractant protein 1 (MCP-1); oxygen radical absorbance capacity (ORAC); total soluble phenolic compounds (TSPC).

## Supplementary Materials

Supplementary material can be found at: https://www.mdpi.com/2076-3921/9/10/984/s1. Table S1: Origin, working pH, and temperature range and declared enzymatic activities of 13 commercial glycosidases. Table S2: Cellulase (CELase), endo-1,4-β-xylanase (XYLase), α-l-arabinofuranosidase (AFase), and feruloyl esterase (FEase) activities in 13 commercial food-grade glycosidases. Table S3: Identification of ferulic acid derivatives in enzymatic hydrolysates of wheat bran by high-performance liquid chromatography coupled to electrospray ionization and quadrupole time-of-flight mass spectrometry (HPLC–ESI–QTOF–MS), including retention time (R.T.), *m*/*z* experimental and calculated, tolerance, error, score, and molecular formula. Table S4: Pearson’s correlation matrix of parameters across different WB enzymatic treatments. Table S5: Pearson’s correlation matrix of parameters across different experimental conditions of WB enzymatic treatments. Figure S1: Scatterplot of correlation analysis between solubilization yield (Ys) and monosaccharide content of WB. Figure S2: Kinetics of FA release from WB treated by Ultraflo XL. Figure S3: Response surface 3D contour plots for combined effects of the two main factors influencing response variables. The third factor was fixed at the optimum level for each response. TSPC (**a**), FA (**b**), ORAC (**c**), DPPH (**d**), FRAP (**e**), MCP-1 (**f**). Abbreviations: 2,2-diphenyl-1-picryl-hydrazyl-hydrate (DPPH); ferric reducing antioxidant power (FRAP); ferulic acid (FA); monocyte chemoattractant protein 1 (MCP-1); oxygen radical absorbance capacity (ORAC); total soluble phenolic compounds (TSPC). Figure S4: Desirability surface 2D contours.
