# Supplementary material for: Enzyme Selection and Hydrolysis under Optimal Conditions Improved Phenolic Acid Solubility, and Antioxidant and Anti-Inflammatory Activities of Wheat Bran
Source: Antioxidants (Basel). 2020 Oct 13;9(10):984. doi: 10.3390/antiox9100984 (PMC7602008; doi:10.3390/antiox9100984)
Supplement: Supplementary file 1 [file antioxidants-09-00984-s001.pdf]

Table S1. Origin, working pH and temperature (T) range and declared enzymatic activities of thirteen commercial glycosidases.

Enzyme	Origin	Working pH	Working T (°C)	Declared activities
Depol 333MDP	*Trichoderma reese*	4-6	50-60	Pentosanase 11,000 U/g (mainly xylanase)
Depol 40L	*Trichoderma* sp./ *Aspergillus* sp.	4-6	40-60	Cellulase 1,200 U/g, pectinase (endogalacturonase) 800 U/g
Depol 667P	*Trichoderma* sp.	5-7	45-55	β-glucanase 12,000 U/g
Depol 670L	Blend of fungi	4-6	50-65	Cellulase 1,200 U/g, Pectinase (endogalacturonase) 800 U/g, feruloyl esterase
Depol 686L	*Trichoderma* sp.	3-6.5	50-65	β-glucanase 5,000 U/g (mainly xylanase)
Depol 740L	*Trichoderma* sp./ *Humicola* sp.	4-6	40-65	β-glucanase 945 U/g and feruloyl esterase
Depol 761P	*Bacillus subtillis*	5.5-6.5	45-55	Xylanase 880 U/g
Depol 793L	*Aspergillus* sp./ *Trichoderma* sp.	4-7	30-60	β-glucanase > 5,500 U/g, pectin lyase > 5000 U/g, cellulase 1,200 U/g
Pectinase 62L	*Aspergillus* sp.	3-5	10-55	Endogalacturonase 2,200 U/g, pectin lyase and arabinofuranosidase
Shearzyme Plus	*Aspergillus oryzae*/ *Trichoderma reesei*	4-5.5	25	Cellulase 350 EGU/g, xylanase 250 FXU-S/g, β-glucanase
Ultraflo L	*Humicola insolens*	4-6	50-65	Endoglucanase 45 FBG/g (xylanase, cellulase, pentosanase, arabinofuranosidase)
Viscoferm	*Aspergillus aculeatus*	3.5	50	Endoglucanase 45 FBG/g (xylanase, cellulase)
Viscozyme L	*Aspergillus aculeatus*	3.5-5.5	25-55	Endoglucanase 100 FBG/g (xylanase, cellulase and hemicellulase)

Table S2. Cellulase (CELase), endo-1,4-β-xylanase (XYLase), *α*-L-arabinofuranosidase (AFase) and feruloyl esterase (FEase) activities in thirteen commercial food-grade glycosidases.

**Enzyme**	**CELase (U/g)** [1]	**XYLase (U/g)**[2]	**AFase (U/g)**[3]	**FEase (mU/g)**[4]
Depol 333MDP	7.02 ± 0.28 ^{d}	16.87 ± 0.20 ^{e}	0.03 ± 0.01 ^{j}	nd
Depol 40L	23.25 ± 1.71 ^{ab}	18.87 ± 0.64 ^{c}	1.72 ± 0.03 ^{d}	nd
Depol 667P	19.14 ± 0.54 ^{c}	20.21 ± 0.57 ^{b}	0.25 ± 0.02 ^{h}	nd
Depol 670L	22.50 ± 0.65 ^{b}	19.13 ± 0.33 ^{c}	3.07 ± 0.07 ^{c}	44.34 ± 9.93 ^{d}
Depol 686L	23.53 ± 0.80 ^{a}	21.93 ± 0.28 ^{a}	0.79 ± 0.03 ^{f}	90.59 ± 6.65 ^{c}
Depol 740L	23.43 ± 0.39 ^{ab}	17.94 ± 0.60 ^{d}	0.35 ± 0.02 ^{g}	290.45 ± 58.48 ^{a}
Depol 761P	nd	17.76 ± 0.39 ^{d}	0.01 ± 0.02 ^{j}	nd
Depol 793L	23.98 ± 0.50 ^{a}	19.91 ± 0.31 ^{b}	1.69 ± 0.04 ^{d}	nd
Pectinase 62L	3.02 ± 0.13 ^{e}	7.31 ± 0.11 ^{g}	3.15 ± 0.08 ^{b}	nd
Shearzyme	23.36 ± 0.51 ^{ab}	16.73 ± 0.41 ^{e}	0.19 ± 0.01 ^{i}	nd
Ultraflo L	23.07 ± 0.50 ^{ab}	16.87 ± 0.13 ^{e}	1.04 ± 0.02 ^{e}	175.39 ± 9.19 ^{b}
Viscoferm	23.26 ± 0.54 ^{ab}	12.93 ± 0.30 ^{f}	0.16 ± 0.02 ^{i}	nd
Viscozyme	3.48 ± 0.15 ^{e}	1.84 ± 0.04 ^{h}	3.41 ± 0.07 ^{a}	nd

Data are the mean ± standard deviation of two replicates. Different superscript letters within a column are significantly different ($p < 0.05$, Duncan's test). [1] One Unit (U) of cellulase activity is defined as the amount of enzyme required to release one μmole of 2-chloro-4-nitrophenol from CellG3 in one minute at pH 6 and 40 °C. [2] One Unit (U) of endo-1,4-β-xylanase activity is defined as the amount of enzyme required to release one μmole of *p*-nitrophenol from the XylX6 in one minute at pH 6 and 40 °C. [3] One Unit (U) of *α*-L-arabinofuranosidase activity is defined as the amount of enzyme required to release one μmole of *p*-nitrophenol (*p*NP) per minute from *p*-nitrophenyl-*α*-L-arabinofuranoside (5 mM) in sodium acetate buffer (100 mM) at pH 6.0 and 40 °C. [4] One Unit (U) of feruloyl esterase activity is defined as the amount of enzyme required to release one μmole of ferulic acid from *p*-nitrophenyl *trans*-ferulate in one minute at pH 6 and 40 °C. [5] nd: not detected.

Table S3. Identification of ferulic acid derivatives in enzymatic hydrolysates of wheat bran by high-performance liquid chromatography coupled to electrospray ionization and quadrupole time-of-flight mass spectrometry (HPLC-ESI-QTOF-MS) including retention time (R.T.), *m/z* experimental and calculated, tolerance, error, score and molecular formula.

Peak	Compound	R.T. (min)	Selected ion	*m/z* calculated	*m/z* experimental	Tolerance (ppm)	Error (ppm)	Score (%)	Molecular formula	UV absorption (nm)
1	FA	15.1	[M − H]−	193.0506	193.0616	10	0.6	99.0	$C_{10}H_{10}O_4$	236, 321
3	DFA i1	15.6	[M − H]−	385.0929	385.1095	10	0.4	93.9	$C_{20}H_{18}O_8$	246, 312
4	DFA i2	19.9	[M − H]−	385.0929	385.0958	10	7.5	94.5	$C_{20}H_{18}O_8$	246, 312
5	DFA dc	20.1	[M − H]−	341.1293	341.1093	10	0.8	95.8	$C_{19}H_{18}O_6$	246, 312
6	VFA	21.6	[M − H]−	343.0823	343.1099	10	0.8	98.8	$C_{18}H_{16}O_7$	236, 321

Abbrebiations: descarboxylated form of dihydroferulic acid (DFA dc); dihydroferulic acid isomer 1 (DFA i1); dihydroferulic acid isomer 2 (DFA i2); ferulic acid (FA); minutes (min); vanillin ferulic acid (VFA).

Table S4. Pearson's correlation matrix of parameters across different WB enzymatic treatments.

	XYLase	AFase	FEase	GLU	GAL	XYL	ARA	TSPC	FA	VFA	DFAi1	DFAdc	DFAi2
CELase	0.602 *	-0.222	0.374	-0.143	-0.632 *	0.549	0.521	0.543	0.585 *	0.372	0.271	0.251	0.429
XYLase		-0.557 *	0.226	-0.721**	-0.767 **	0.462	0.257	0.460	0.384	0.235	0.126	0.085	0.281
AFase			-0.176	0.535	0.588 *	-0.547	−0.132	-0.372	0.004	-0.049	-0.084	-0.049	-0.051
FEase				0.015	-0.325	0.049	0.915 ***	0.306	0.756 **	0.689 **	0.535	0.505	0.822 **
GLU					0.727 **	-0.375	0.032	-0.209	-0.030	0.040	0.071	0.097	0.010
GAL						-0.723 **	-0.487	-0.596 *	-0.541	-0.411	-0.328	-0.298	-0.449
XYL							0.278	0.614 *	0.246	0.117	0.095	0.075	0.118
ARA								0.481	0.908 ***	0.822 ***	9.664 *	0.640 *	0.919 ***
TSPC									0.552	0.617 *	0.657 *	0.638 *	0.514
FA										0.869 ***	0.753 **	0.732 **	0.954 ***
VFA											0.953 ***	0.942 ***	0.965 ***
DFA i1												0.998 ***	0.854 ***
DFA dc													0.835 ***

Data represent the determination coefficients (*r*). Significance indicators: * $p \leq 0.05$, ** $p \leq 0.01$ and *** $p \leq 0.001$. Abbreviations: α-L-arabinofuranosidase (AFase); arabinose (ARA); cellulase (CELase); endo-1,4-β-xylanase (XYLase); descarboxylated form of dihydroferulic acid (DFA dc); dihydroferulic acid isomer 1 (DFA i1); dihydroferulic acid isomer 2 (DFA i2); ferulic acid (FA); feruloyl esterase (FEase); galactose (GAL); glucose (GLU); total soluble phenolic compounds (TSPC); vanillin ferulic acid (VFA); xylose (XYL).

Table S5. Pearson's correlation matrix of parameters across different experimental conditions of WB enzymatic treatments.

	TSPC	FA	ORAC	ABTS	DPPH	FRAP	MCP1	IL6	TNFα
Ys	-0.286	0.803 ***	-0.102	-0.239	-0.348	-0.318	-0.247	0.321	-0.485
TSPC		-0.404	0.828 ***	0.866 ***	0.795 ***	0.920 ***	-0.078	-0.133	-0.230
FA			-0.365	-0.338	-0.328	−0.400	0.099	0.257	0.007
ORAC				-0.325	0.049	0.915 ***	0.306	0.756 **	0.689 **
ABTS					0.835 ***	0.920 ***	-0.008	-0.143	-0.184
DPPH						0.929 ***	0.081	0.038	0.054
FRAP							0.042	-0.076	-0.074
MCP-1								0.034	0.783
IL-6									-0.049

Data represent the determination coefficients (r). Significance indicators: *** $p \leq 0.001$. Abbreviations: 2,2′-azino-bis(3-ethylbenzothiazoline-6-sulfonic acid) (ABTS); 2,2-diphenyl-1-picryl-hydrazyl-hydrate (DPPH); ferric reducing antioxidant power (FRAP); interleukin 6 (IL-6); monocyte chemoattractant protein 1 (MCP-1); oxygen radical absorbance capacity (ORAC); wheat bran solubilization yield (Ys); tumor necrosis factor α (TNF-α).

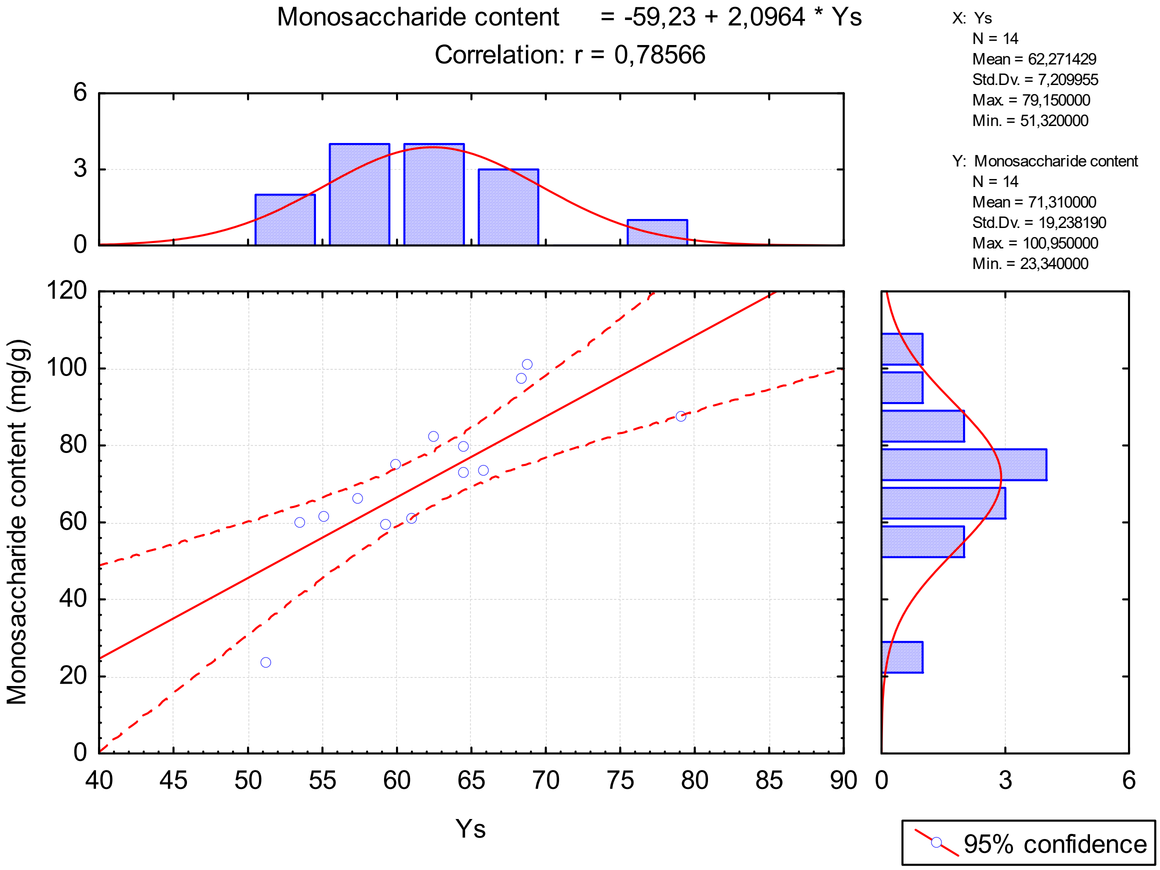

Figure S1. Scatterplot of correlation analysis between solubilisation yield (Ys) and monosaccharide content of WB.

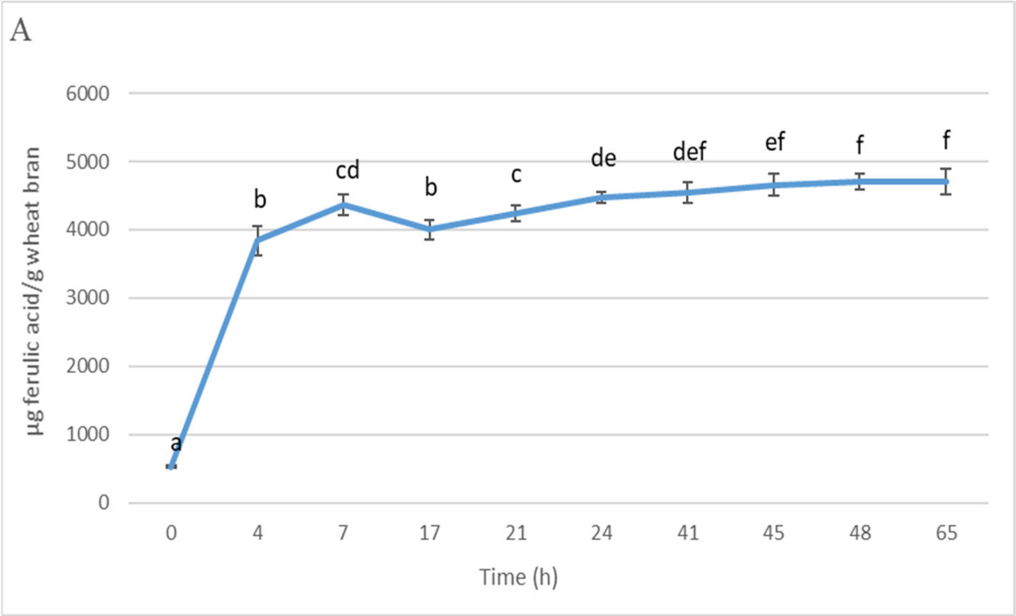

Figure S2. Kinetics of FA release from WB treated by Ultraflo L.

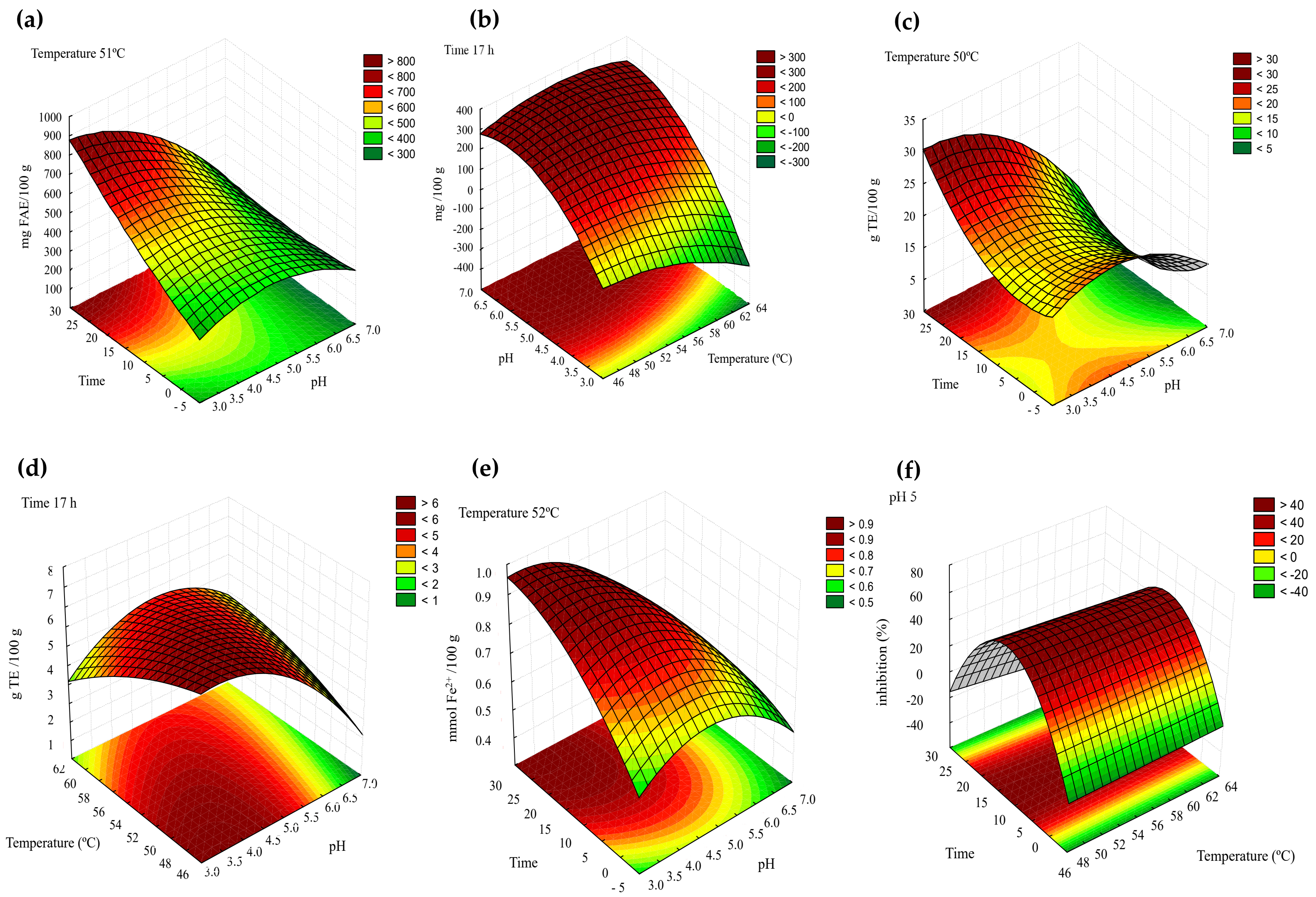

Figure S3. Response surface 3D contour plots for combined effects of the two main factors influencing response variables. The third factor was fixed at the optimum level for each response. TSPC (**a**), FA (**b**), ORAC (**c**), DPPH (**d**), FRAP (**e**), MCP-1 (**f**). Abbreviations: 2,2-diphenyl-1-picryl-hydrazyl-hydrate (DPPH); ferric reducing antioxidant power (FRAP); ferulic acid (FA); monocyte chemoattractant protein 1 (MCP-1); oxygen radical absorbance capacity (ORAC); total soluble phenolic compounds (TSPC).

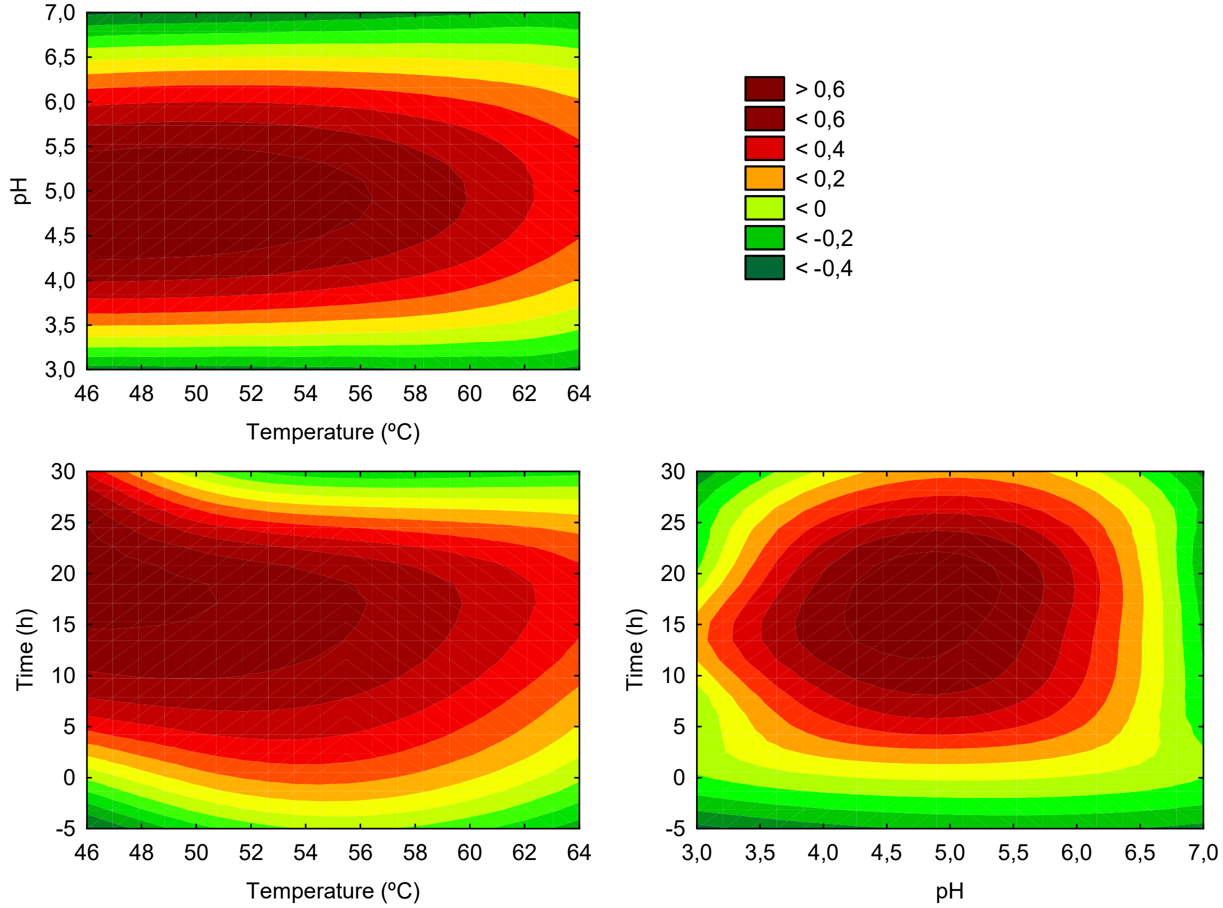

Figure S4. Desirability surface 2D contours.
